# High plasma salivary α-amylase, but not high AMY1 copy number, associated with low obesity rate in Qatari adults: cross-sectional study

**DOI:** 10.1038/s41598-020-74864-6

**Published:** 2020-10-21

**Authors:** Neyla Al-Akl, Richard I. Thompson, Abdelilah Arredouani

**Affiliations:** 1 Qatar Biomedical Research Institute, Hamad Bin Khalifa University, Doha, Qatar; 2 College of Health and Life Sciences, Hamad Bin Khalifa University, Doha, Qatar

**Keywords:** Enzymes, Obesity, Risk factors, Predictive markers

## Abstract

The relationship between salivary α-amylase activity (psAAa) or AMY1 copy number and the risk of obesity remains controversial. We aimed to assess this relationship in a cohort from Qatar, where obesity affects 43% of adults. The relationship was investigated cross-sectionally in 923 Qatari adults from the Qatar biobank cohort. AMY1 CN was estimated form whole genome sequencing data. The associations with obesity prevalence were assessed by linear and logistic regressions. We found no difference in AMY1 CN between obese and normal-weight individuals. However, the psAAa was significantly lower in obese individuals. Significant inverse correlations were found between adiposity markers and psAAa in both sexes, but were marginally stronger in men. A significant effect of high psAAa, but not AMY1 CN, on reduced obesity rates was identified in men (OR per psAAa unit 0.957 [95% CI 0.937–0.977], *p* < 0.001, with psAAa ranging between 5 to 66 U/L). A significantly higher prevalence of obesity was observed in the lowest quartile of psAAa in men (75% (Q1) vs. 36% (Q4), *p* < 0.001) and women (74% (Q1) vs 56% (Q4), *p* = 0.009). Our findings suggest that high psAAa, but not AMY1 CN, has a potential positive benefit against obesity in the Qatari population.

## Introduction

The salivary α-amylase (sAA) initiates the digestion in the mouth of large α-linked polysaccharides, such as starch or glycogen, to yield a mixture of di-saccharides, tri-saccharides and some glucose^1^. It is produced predominantly by the salivary glands, and represents at least 50% of total saliva protein content^2^. The concentration of sAA in saliva is determined by several environmental factors, including stress^3^, exercise^4^ and circadian rhythms^5^. In humans, the sAA is encoded by the AMY1 gene, and its production is influenced by the copy number variations (CNVs) in this gene. AMY1 is one of the most variable loci in the human genome, ranging from 2 to 20 diploid copies^6–9^; and both saliva and serum levels of sAA positively correlate with the number of copies of the AMY1 gene^6–8,10^. Variability in both *AMY1* CNVs and sAA protein levels has been observed among human populations, with those traditionally consuming starch-rich diets showing higher AMY1 gene CNV and sAA levels than those consuming low-starch diets^8,11^. Several population studies on both adults and school children identified a negative correlation between AMY1 gene CN and common obesity; low AMY1 gene CN was associated with increased body mass index (BMI) and waist circumference^7,12–21^. However, other studies reported no association^22–24^ or a positive association between AMY1 gene CN and BMI^25^. Furthermore, AMY1 gene CN inversely correlated with high insulin resistance (IR) in a study of asymptomatic Korean men^26^, but no association was found with IR in a recent study^13^. Very recently, low AMY1 gene CN was also associated with increased cardiovascular disease risk and inflammation in overweight or obese adults^27^. Basal sAA activity was also reported to be negatively associated with behavioral preference for foods high in sugar^28^, which may explain the predisposition of low amylase subjects to obesity^7^. Moreover, low total serum amylase levels (pancreatic + salivary) were significantly associated with moderate to severe non-alcoholic fatty liver disease (NAFLD) in asymptomatic adults independent of metabolic syndrome, diabetes and obesity^29^. Also, levels of serum amylase were significantly lower in NAFLD patients with metabolic syndrome (MetS) as compared with NAFLD patients without MetS and healthy controls^30^. All together, these studies show that, contrary to the classical view that obesity genes are involved in appetite regulation and are expressed mainly in the brain^31^, there exist a genetic link between carbohydrate metabolism and obesity and that sAA may play a role in this link. Moreover, the observations above also suggest that the sAA activity could serve as a potential marker to predict metabolic disorders, particularly in populations where starch is a staple food.

In most of the above-mentioned studies, the link between sAA and metabolic disorders was suggested by investigating the association of the different obesity markers, such as BMI or insulin resistance, with the AMY1 gene CN, which was determined by different techniques. However, a recent study showed that AMY1 CN does not explain the majority of the observed variation in expression and activity between individuals^9^. The same study also showed that *AMY1*-odd and *AMY1*-even haplotypes showed a different, though not statistically significant (*p* = 0.052), relationship between copy number and expression levels. Of note, plasma sAA activity is ultimately the end-product of the expression of AMY1 gene and is the factor that has the greatest influence on the degradation of polysaccharides. These observations prompted us to investigate the association between obesity markers and plasma sAA activity in adults in Qatar, where obesity prevalence is one of the highest in the world and starch, in the form of rice, is a staple food.

## Results

### Baseline characteristics of the study participants

**Figure 1:**
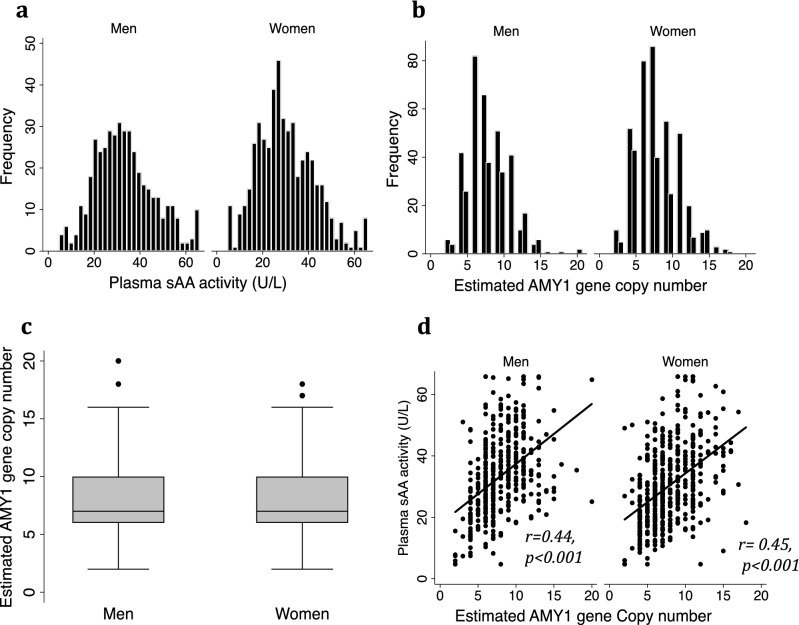
Distribution and correlation of psAAa and AMY1 CN by gender. (**a**) Distribution pf plasma sAA activity and (**b**) AMY1 gene CN in men (n = 431) and women (n = 498). (**c**) Box and whiskers plots of AMY1 gene CN by gender (**d**) Correlation between plasma sAA activity and AMY1 gene CN by gender.

The baseline characteristics of the study participants (53.6% women) are shown in Table 1. The mean age was 39 ± 11 for men (n = 431) and 39.9 ± 13 years for women (n = 498). There were significant differences between men and women in almost all adiposity markers tested, including waist and hip circumferences (WC and HC), waist to hip ratio (WHR), fat mass (FM), fat mass index (FMI), body fat percentage (BF%), body adiposity index (BAI), Visceral Adipose tissue mass (VAT), and body weight (BW) (Table 1). Furthermore, 38% of men and 46% of women were obese (BMI ≥ 30 kg/m^2^); while 11.6% of men and 12.8% of women had T2D (Hb1Ac ≥ 6.5%). The plasma sAA activity (psAAa) ranged between 4.73 and 65.82 U/L (Fig. 1a), and the mean psAAa was significantly higher in men (33.2 ± 12.4 U/L vs. 30.08 ± 12.4 U/L; *p* < 0.001) (Table 1). The significant differences observed between men and women in many adiposity markers as well as in mean psAAa prompted us to analyze the relationship between the different adiposity features and either psAAa or AMY1 CN separately by gender.

**Table 1:** Baseline characteristics of the participants by gender.

Variables	Male (n = 431)	Female (n = 498)	*p* value
Age (years)	39.0 (11.3)	39.9 (12.9)	0.25
AMY1 CNV (median)	7	7	0.47
psAAa (U/L)	33.2 (12.8)	30.08 (12.4)	**< 0.001**
Obesity (%)	38	46	**0.01**
Diabetes (%)	11.6	12.8	0.56
BMI (kg/m^2^)	29.1 (5.5)	29.6 (6.3)	0.21
Waist (cm)	95.0 (13.1)	84.6 (14.1)	**< 0.001**
Hip (cm)	105.7 (10.4)	107.9 (11.7)	**0.003**
WHR	0.9 (0.1)	0.8 (0.1)	**< 0.001**
Fat mass (kg)	23.9 (8.9)	29.9 (11.4)	**< 0.001**
FMI	8.3 (3.1)	12.0 (4.5)	**< 0.001**
Body fat (%)	27.9 (6.7)	39.3 (7.8)	**< 0.001**
BAI	28.9 (4.7)	36.5 (6.2)	**< 0.001**
VAT (kg)	1.4 (0.8)	0.9 (0.6)	**< 0.001**
BW(kg)	85.5 (16.8)	73.3 (16.3)	**< 0.001**

Values are presented as mean ± SD, median or proportion. BAI: Body adiposity index; BMI: body mass index; BW: Body weight; FMI: fat mass index; psAAa: plasma salivary alpha-amylase activity; WHR: waist-to-hip ratio; VAT: visceral adipose tissue. Significant *p* values at 5% level are in bold.

### Correlation between plasma sAA activity and AMY1 gene CN

**Figure 2:**
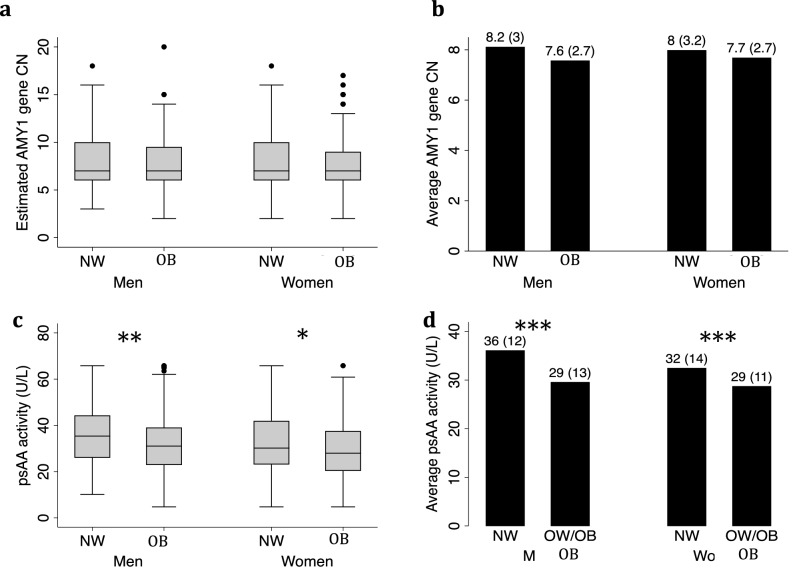
Mean and Median of psAAa and AMY1 CN by gender. (**a**) Median and (**b**) mean of estimated AMY1 gene CN in normal-weight (NW, n = 103 for men and n = 113 for women) and obese (OB, n = 164 for men and n = 231 for women) individuals by gender. (**c**) Median and (**d**) mean of plasma sAA activity (**a**) in NW and OB individuals by gender. Significance: ***p < 0.001; **p < 0.01; *0.5.

We have estimated the AMY1 gene copy number for all the participants from whole genome sequencing data and found extensive variation in AMY1 gene copy number in both sexes, ranging from 2 to 20 (Fig. 1b). The ratio of odd to even AMY1 gene CN was 1.04 in men and 0.98 in women. In both sexes, 90% of the individuals had AMY1 gene CN between 4 and 12. The median of AMY1 gene CN was 7 in both sexes (Fig. 1c). Further, we found a moderate but significant positive correlation between AMY1 gene CN and psAAa in both sexes (r = 0.44; *p* < 0.001 for men, and r = 0.45; *p* < 0.001 for women) (Fig. 1d). The association was further assessed with linear regression adjusted for age. In men and women respectively, each additional AMY1 gene copy increased the psAAa by 1.97 U/L [95% CI 1.6–2.3] and 1.88 U/L [95% CI 1.5–2.2]; and explaining 20% of the respective activity. We also used linear regression to assess the association between psAAa and AMY1 gene CN in obese men and women and found that a single AMY1 gene copy influences psAAa more in men (β coefficients; 2.12 U/L vs. 1.52 U/L respectively for men and women). Within each sex, we compared the median and the mean of AMY1 gene CN between normal weight (NW) (BMI < 25 kg/m^2^) and obese individuals (OB) (BMI ≥ 30 kg/m^2^) and found no significant difference (Fig. 2a,b). Interestingly, however, when we looked at the psAAa we found that the median and the mean of psAAa were both significantly higher in NW compared to obese individuals (Fig. 2c,d).

### Differences in demographic, clinical, and biochemical characteristics between psAAa groups

The median values of psAAa were used to define low and high psAAa groups. Men with psAAa < 32U/L were considered as low psAAa (LpsAAa_m), while those with psAAa ≥ 32U/L were considered high psAAa (HpsAAa_m). Likewise, women with psAAa < 28 U/L were considered low psAAa (LpsAAa_w), while those with psAAa ≥ 28U/L were considered high psAAa (HpsAAa_w). There were significantly more obese individuals in LpsAAa_m than in HpsAAa_m group (70% vs. 51%; *p* = 0.002). In women, however, the percentage of obese subjects was not significantly different between LpsAAa_w and HpsAAa_w groups (71% vs. 63%; *p* = 0.16). Additionally, in men, BMI, WC, HC, FM, FMI, BF%, BAI, VAT and BW, were all significantly (*p* < 0.05) higher in the LpsAAa_m group (Table 2). In contrast, we did not see any significant differences for any the above markers in women psAAa groups (Table2).

**Table 2:** The demographic, clinical, and biochemical characteristics of the participants within high and low psAA activity by gender.

	Men			Women		
	LpsAAa (n = 214)	HpsAAa (N = 217)	*p* value	LpsAAa (n = 259)	HpsAAa (N = 239)	*p* value
Age (years)	39.4 (11.1)	38.6 (11.5)	0.49	39.4 (12.2)	40.5 (13.6)	0.32
psAAa (U/L)	23 (6.3)	43.3 (9.1)	**< 0.001**	20.6 (6.1)	40.4 (8.9)	**< 0.001**
BMI (kg/m^2^)	30.1 (5.8)	28.1 (5.0)	**< 0.001**	29.8 (6.4)	29.3 (6.3)	0.35
Waist (cm)	96.7 (13.2)	93.3 (12.8)	**0.007**	84.8 (13.6)	84.4 (14.6)	0.78
Hip (cm)	107.2 (10.9)	104.2 (9.6)	**0.003**	108.4 (11.4)	107.4 (12.0)	0.35
WHR	0.9 (0.1)	0.9 (0.1)	0.25	0.8 (0.1)	0.8 (0.1)	0.66
Fat mass (kg)	25.3 (9.2)	22.5 (8.5)	**< 0.001**	30.7 (11.3)	29.0 (11.4)	0.098
FMI (kg/m^2^)	8.8 (3.2)	7.7 (3.0)	**< 0.001**	12.2 (4.5)	11.7 (4.5)	0.23
Body fat (%)	28.9 (6.9)	26.9 (6.4)	**0.002**	39.8 (7.5)	38.7 (8.1)	0.13
BAI index	29.5 (5.1)	28.2 (4.2)	**0.005**	36.6 (6.1)	36.5 (6.3)	0.85
VAT mass (kg)	1.4 (0.8)	1.3 (0.7)	**0.031**	0.9 (0.6)	0.9 (0.6)	0.16
BW (kg)	88.1 (17.3)	82.9 (16.0)	**0.001**	74.2 (16.2)	72.2 (16.4)	0.17
Obesity (%)	70	51	**0.002**	71	63	0.16

Values are presented as mean ± SD or proportion. Significant p values at 5% level are in bold.

### Differences in demographic, clinical, and biochemical characteristics between AMY1 gene CN groups

As mentioned above, the AMY1 gene CN median in our cohort was 7. This value was used to define groups of low (< 7copies) and high (≥ 7 copies) AMY1 gene CN groups. Interestingly, despite the significant difference in the AMY1 gene CN median between the groups in both sexes, and in contrast to low and high psAAa groups, only FMI and BAI indexes were significantly higher in the group of men with low AMY1 gene CN (Table 3).

**Table 3:** The demographic, clinical, and biochemical characteristics of the participants within high and low AMY1 CN groups by gender.

Variables	Men			Women		
	Low AMY1 CN (n = 160)	High AMY1 CN (N = 271)	*p* value	Low AMY1 CN (n = 190)	High AMY1 CN (N = 308)	*p* value
Age (years)	40.7 (11.5)	38.0 (11.1)	0.014	39.4 (12.6)	40.2 (13.1)	0.48
AMY1 CN (median)	5.1	9.5	**< 0.001**	4.9	9.5	**< 0.001**
BMI (kg/m^2^)	29.6 (5.8)	28.8 (5.3)	0.11	29.5 (5.9)	29.6 (6.6)	0.91
Waist (cm)	96.1 (13.2)	94.3 (13.0)	0.17	84.4 (13.7)	84.7 (14.3)	0.84
Hip (cm)	106.4 (11.1)	105.3 (10.0)	0.26	107.9 (10.6)	107.9 (12.3)	0.98
WHR	0.9 (0.1)	0.9 (0.1)	0.23	0.8 (0.1)	0.8 (0.1)	0.77
Fat mass (kg)	24.9 (9.4)	23.3 (8.7)	0.065	30.2 (10.2)	29.7 (12.1)	0.63
FMI (kg/m^2^)	8.7 (3.2)	8.0 (3.1)	**0.032**	12.0 (4.1)	11.9 (4.8)	0.80
Body fat (%)	28.5 (6.4)	27.5 (6.9)	0.16	39.7 (7.0)	39.0 (8.3)	0.32
BAI	29.5 (5.0)	28.5 (4.4)	**0.031**	36.5 (5.8)	36.6 (6.4)	0.91
VAT mass (kg)	1.4 (0.7)	1.3 (0.8)	0.47	0.9 (0.6)	0.9 (0.6)	0.55
BW (kg)	86.3 (17.7)	85.0 (16.3)	0.43	73.3 (15.3)	73.3 (17.0)	0.98

Values are presented as mean ± SD. Significant p values at 5% level are in bold.

### Relationship between adiposity markers and plasma sAA activity

**Figure 3:**
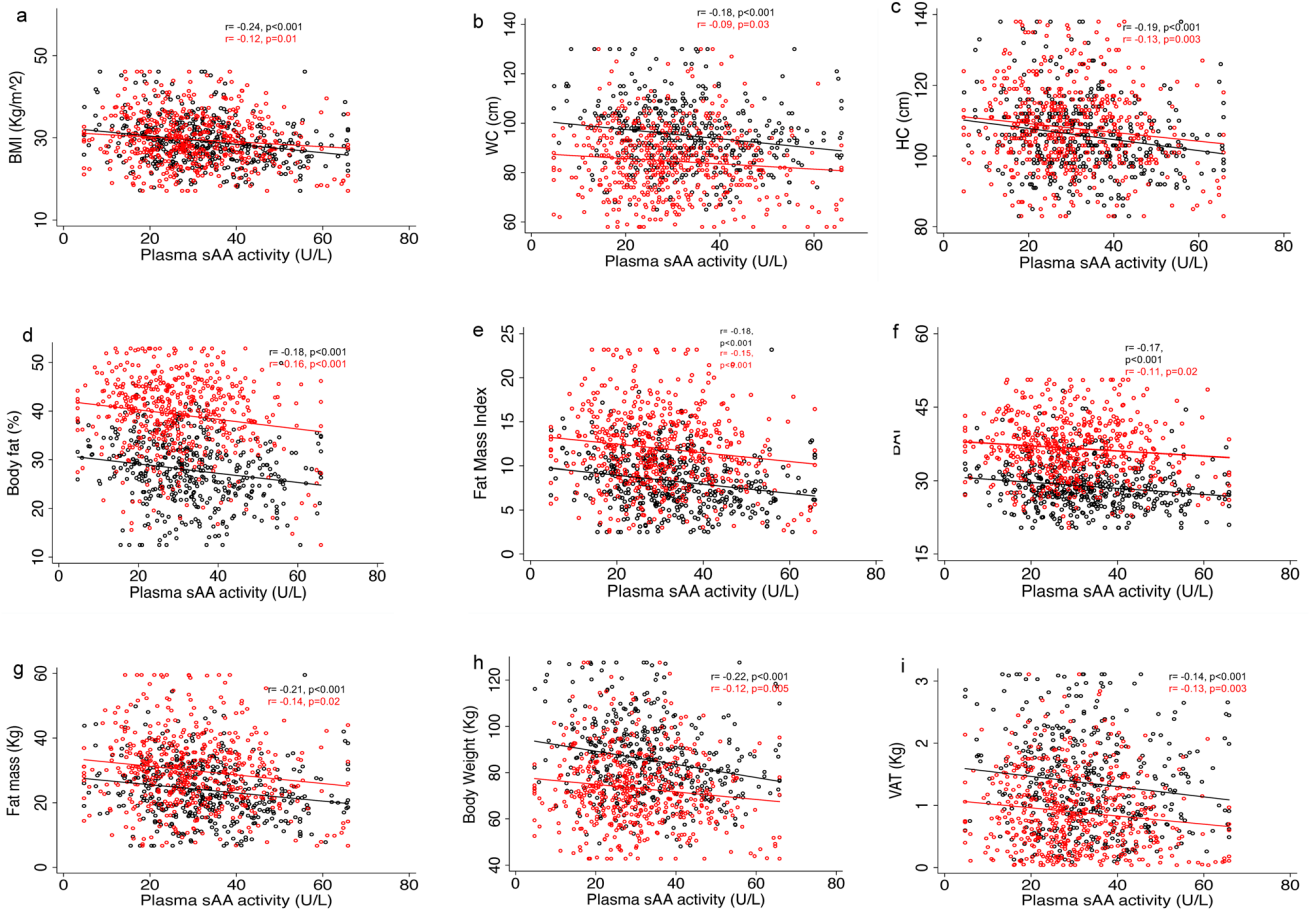
Scatter plots and best fit lines depicting correlations between psAAa and different adiposity markers including BMI (**a**), WC (**b**), HC (**c**), BF% (**d**), Fat mass (**e**), BAI (**f**), FMI (**g**), BW (**h**) and VAT (**i**) in men (open circles and solid lines) and women (open squares and dotted lines). r and p values in bold are for men.

To assess the relationship between psAAa and different adiposity markers in men and women we first ran Pearson's correlations (supplementary table S1). Except for WHR, significant, though weak, inverse correlations between psAAa and all the other parameters tested, including BMI, WC, HC, fat mass, FMI, BF%, BAI, VAT and BW were observed in both sexes (supplementary table S1 and Fig. 3). Interestingly, when we tested the relationship between AMY1 gene CN and the same parameters no significant correlations were found in women; while in men significant negative correlations, though weaker than for psAAa, were found only for BMI, fat mass, FMI, BF%, and BAI (supplementary table S1). To further investigate the association between the psAAa and the adiposity markers that displayed significant correlation coefficients that are ≥  + 0.1 or ≤ − 0.1, we used linear regression adjusted for age (Table 4). Hence, the variables BMI, waist, hip, fat mass, FMI, BF%, BAI, VAT, and BW all remained significantly and inversely associated with psAAa in both sexes (Table 4). The highest decrease per 1 unit increase in psAAa in men was observed for BW (β = − 0.28) followed by WC (β = − 0.19) and HC (β = − 0.16). In women, the highest decrease per 1 unit increase in psAAa was observed for BW (β = − 0.16) followed by fat mass (β = − 0.13) and HC (β = − 0.12).

**Table 4:** Linear regression adjusted for age examining associations between psAAa and different adiposity markers.

Dependent variables	Men (n = 431)	Women (n = 498)
	β (95% CI)	β (95% CI)
BMI (kg/m^2^)	− 0.10 (− 0.14 to 0.06)***	− 0.06 (− 0.09 to 0.02)*
Waist (cm)	− 0.19 (− 0.28 to 0.10)***	− 0.10 (− 0.18 to 0.21)*
Hip (cm)	− 0.16 (− 0.23 to 0.08)***	− 0.12 (− 0.20 to 0.04)*
Fat mass (kg)	− 0.13 (− 0.19 to .006)***	− 0.13 (− 0.20 to 0.006)***
FMI (kg/m^2^)	− 0.05 (− 0.07 to 0.03)***	− 0.04 (− 0.07 to 0.02)*
BF (%)	− 0.09 (− 0.14 to 0.04) ***	− 0.09 (− 0.14 to 0.05)***
BAI	− 0.06 (− 0.09 to 0.02)***	− 0.05 − 0.09 to 0.01)*
VAT (kg)	− 0.008 (− 0.013 to 0.003) *	− 0.006 (− 0.01 to 0.002)*
BW (kg)	− 0.28 (− 0.40 to 0.16)***	− 0.16 (− 0.26 to 0.05)*

We then used adjusted logistic regression to test the association between psAAa and rate of obesity in a binary case–control (obese/normal-weight) framework. We found a significant association between high psAAa and lower rate of obesity in men (OR 0.957; 95% CI 0.93–0.977; *p* < 0.001; n = 267) but not in women (OR 0.98, 95% CI 0.96–1.002; *p* = 0.07; 267 n = 344). No significant association was observed between AMY1 gene CN and rate of obesity (Table 5). Given that some individuals with diabetes may take medication that can cause weight gain, we ran the same logistic regression analysis by excluding all the subjects with diabetes (i.e. when HbA_1c_ ≥ 6.5%), or by adjusting for the diabetes status and we did not see any difference (data not shown).

**Table 5:** Association between psAAa or AMY1 gene CN and the rate of obesity in men and women.

Predictor variables	Men (n = 267)				Women (n = 344)			
	Cases (n = 164)	Controls (n = 103)	OR (95% CI)	*p* value	Cases (n = 231)	Controls (n = 113)	OR (95% CI)	*p* value
psAAa (U/L)	29.5 (12.67)	36.1 (12.37)	0.957 (0.937–0.977)	**< 0.001**	28.71 (11.40)	32.47 (14.32)	0.979 (0.96–1.002)	0.07
AMY1 CN	7	7	0.950 (0.875–1.032)	0.221	7	7	0.943 (0.858–1.035)	0.232

Data are mean ± SD. The table includes the logistic regression analysis adjusted for age. Cases are the obese individuals while the controls are the normal-weight.

Similar results were obtained when the overweight and obese individuals were pooled together as cases (supplementary table S2). Subsequently, we estimated the prevalence of obesity in psAAa quartiles and found that the obesity rates were significantly higher in the lowest as compared to the highest psAAa quartile in both men (*p* < 0001) and women (*p* = 0.009) (Table 6).

**Table 6:** Prevalence of obesity by sex-specific quartiles of psAAa.

	Men (267)				Women (344)			
	Q1 (68)	Q2 (59)	Q3 (74)	Q4 (66)	Q1 (99)	Q2 (90)	Q3 (80)	Q4 (75)
psAAa (U/L)	16.99 (4.70)	46.47 (2.31)	34.48 (2.66)	49.61 (7.45)	16.15 (4.63)	26.42 (2.10)	34.55 (2.85)	47.87 (7.37)
NW/OB	17/51	21/38	23/51	42/24	25/74	31/59	24/56	33/42
Obesity prevalence (%)	75*	64	68	36*	74*	65	70	56*

NW/OB: ratio of normal-weight over obese individuals. psAAa presented as mean (SD).

*Significant difference at 5% level in obesity prevalence between Q1 and Q4 in men and women.

## Discussion

In the present study, we found that men, but not women, expressing low psAAa had significantly increased adiposity markers, including BMI, WC, HC, fat mass, FMI, fat %, BAI, VAT and BW, as compared to men expressing high psAAa. Interestingly, in men with low AMY1 gene CN, only FMI and BAI were significantly higher compared to men with high AMY1 gene CN. We also observed negative and significant associations between psAAa, but not AMY1 gene CN, and all the adiposity markers tested. On the other hand, despite normal-weight versus obese men and women having equal AMY1 CN median, we found a significantly lower psAAa in the obese individuals. Finally, we report that low psAAa, but not low AMY1 CN, is associated with reduced obesity rate in both sexes. Several studies have investigated the relationship between AMY1 gene CN and predisposition to metabolic disorders, including obesity, T2D and insulin resistance, in both children and adults. However, contradicting results were reported^7,12–15,18,19,21–25,32–34^.The reported discrepancies remain unexplained, and possible reasons include the different techniques used to estimate the AMY1 gene CN^35^ as well as the different ethnicities^21,36^ and age^37^ of the populations studied. The distribution of AMY1 gene CN in our study sample (2–20 copies) is comparable to what was reported previously across modern human populations^38^. The median of AMY1 gene CN was 7, which is like the one reported recently^15,17^. However, other studies previously reported a median of 4^7,14,27^. We have no explanation for this large difference in median AMY1 gene CN, but the different ethnicities studied, or the techniques used to estimate the CNV might play a role. The AMY1 gene CN median did not differ between sexes, and there was a moderate but significant positive correlation between AMY1 gene CN and psAAa in both sexes (Fig. 1d). However, the psAAa was significantly higher in men (Table 1). This observation suggests a possible sexual dimorphism of AMY1 gene expression and/or translation, i.e. some AMY1 gene copies may be less functional in women or more functional in men, with functional consequences with respect to utilization of carbohydrates by men and women. Indeed, we have observed that a single AMY1 gene copy influences psAAa more in obese men than in obese women (β coefficients; 2.12 U/L [95% CI 1.56–2.68] vs. 1.52 U/L [95% CI 1.02–2.02] respectively for men and women), while in normal-weight individuals (BMI < 25 kg/m^2^) it is the opposite (β coefficients; 1.49U/L [95% CI [0.74–2.23] vs. 1.88 U/L [95% CI 1.13–2.64], respectively for men and women). This potential sexual dimorphism warrants further investigations to fully elucidate the underlying mechanism(s). The observation also indicates that, as suggested previously^9^, the copy number variation of human AMY1 is probably not the only factor that determines the variation in salivary α-amylase expression and activity. It additionally questions the belief that more copies of AMY1 gene means more salivary α-amylase activity and, hence, an improved better ability to digest starch^8,38,39^. It is, thus, not straightforward to predict an individual’s psAA activity solely from their AMY1 gene CN, and, therefore, caution must be exercised when studying the effect of AMY1 gene CN, and other genes with CNV for that matter. Our findings also suggest that knowing the AMY1 CN is not sufficient to predict predisposition to obesity without knowing how the AMY1 CN translates in terms of psAA activity. It further raises the question of the generalizability of the associations reported previously between AMY1 gene CN and BMI even within the same population. Contrary to other reports^20^, we found no significant differences in either the mean or the median of AMY1 gene CN between obese and normal-weight men or women in our cohort (Fig. 2a,b). Nevertheless, the obese men and women had significantly lower mean and median psAAa (Fig. 2c,d). This observation again confirms that the predispositions to weight gain is rather attributed to low psAAa regardless of the AMY1 gene CN and may explain, at least partially, why some studies reported no association between AMY1 gene CN and BMI^22–24^. Our observation agrees with a recent study^32^, which found a modest contribution of AMY1A CN to lower BMI but a significant negative contribution of AMY1 activity at baseline to the change in BMI during the 9-year follow-up.

The significantly higher mean values of adiposity markers observed only in men with low psAAa indicate that men with low psAAa are more prone to obesity than women with low psAAa (Table 2) and suggests a potential sex-specific mechanism of action of sAA, probably due to gender-specific factors such as sex hormones or others^40^. The proneness of men with low psAAa to obesity is further confirmed with the higher correlations between psAA and several obesity markers observed in men as compared to women (table S1).

Our findings disagree with a previous work by Viljakainen et al.^20^, which reported a significant inverse correlation between AMY1 gene CN and whole-body fat% or BMI in severely obese young women, while no association was observed in obese males. This discrepancy might in part be explained by the ethnicity of the populations used (Caucasian vs. Middle Eastern), by the difference in the age of the respective populations used (15-25y vs. 18 to 68y), and by the fact that the participants in Viljakainen’s study had a history of childhood severe onset obesity.

In adjusted linear regression examining associations between psAAa and different adiposity markers, the absolute value of β-coefficients of most of the markers, including BMI, WC, HC, VAT, BAI and BW, were higher in men then in women, further indicating the increased susceptibility of men with low psAAa to obesity. Indeed, in men obesity was significantly more prevalent among individuals in the lowest quartile (Q1) of psAAa as compared to the highest quartile (Q4) (Table 6). In concordance, it is only in men that increased levels of psAAa, but not increased AMY1 gene copy number, associated with lower rate of obesity (Table 5).

The mechanisms that link psAAa or AMY1 CN to risk of obesity are not well understood. Recent studies have shown that variation in AMY1 1 gene CN, and consequently sAAa, may contribute significantly to differences in dietary starch intake^39^ and enhance the preference for foods high in sugars^28^. Consequently, in regions, such as the Middle east, where starch is a staple food, and western diets rich in calorie-dense foods and drinks have dominated the food culture in the last decades, individuals, mainly men, with low psAAa might be at greater risk of weight gain because of increased intake of sugary foods. Previously, Mandel and colleagues^41^ demonstrated that normal-weight adults with high sAA activity displayed improved glycemic control upon liquid starch ingestion (but not after glucose load) and suggested that individuals with low sAA activity might be at risk of developing T2D. Given the link between obesity and T2D, the results from our study, along with Mandel’s findings, indicate that high activity of the sAA is potentially beneficial for energy metabolism and weight gain mainly in populations with diets rich in starch, like in the Middle East region. Furthermore, given the role of sAA in the digestion of starch, variation in the activity of this enzyme might influence gut microbiota through dietary carbohydrate processing^16,42^. Lately, low AMY1 gene CN was associated with gut *Prevotella* abundance in Mexican children and adults^16^. *Prevotella* was previously reported to be metabolically favorable^43^. Additionally, an association was reported between AMY1 activity and lactate, a product of complex intestinal carbohydrate fermentation^32^. Also, a mouse genetic linkage study^42^ reported a SNP (rs29982345) close to AMY1 gene that was significantly associated with weight gain and enrichment of genera *Enterobacteriaceae* known to be correlated with obesity in humans^44,45^. Further investigations are needed to fully understand how salivary α-amylase activity might contribute to the modulation of the function of the gastrointestinal tract in humans and directly or indirectly affect food absorption or appetite.

The main limitation of our study is that it is cross-sectional and not prospective. Therefore, we can only report associations. On the other hand, compared to the only study that investigated the association between amylase and obesity in Middle East (n = 200)^33^, our cohort was more than 4 times larger (n = 929) and thus statistically more powerful. Moreover, our data is obtained from a research biobank and the participants are well phenotyped.

In summary, we found that psAAa, but not AMY1 gene CN, is significantly decreased in obese compared to normal-weight men and women. Moreover, significant inverse associations between several adiposity markers and psAAa, but not AMY1 gene CN, were observed in both sexes, but these associations were stronger in men, which suggests that men with low psAAa are more susceptible to weight gain if they consume starch-rich foods chronically. We also report that high psAAa levels, but not high AMY1 CN, are associated with reduced odds of being obese in men but not in women. Finally, our findings imply that AMY1 gene CN is not the only factor that determines the sAA activity in plasma, and more investigations are warranted to better understand the relation between AMY1 gene CN and sAA expression and activity.

## Research design and methods

### Study design and participants

In this cross-sectional study we utilized baseline clinical, anthropometric, demographic data as well as biospecimens of 929 individuals obtained from the Qatar Biobank cohort (QBB), a well-phenotyped cohort that recruits adults (aged > 18 years) from the general population^46^. This study was approved by the local ethics committees, the institutional review boards at both Qatar Biomedical Research Institute (QBRI) (IRB number: 2017-001) and QBB (IRB number: Ex -2017- RES-ACC -0054-0018). All work was performed in compliance with the ethical standards stated by the declaration of Helsinki. All participants gave written informed consent for their data and biospecimens to be used in medical research (https://www.qatarbiobank.org.qa). The inclusion criteria for this study were: (1) being a Qatari citizen; (2) fasting for more than 6 h at the time of collection of biospecimens; (3) availability of whole genome data. Plasma was prepared within 2 h of blood collection. Data and biospecimens were supplied to Qatar Biomedical Research Institute in an anonymous coded manner.

### Anthropometric and clinical measures

All clinical measurements were carried out by the central laboratory in Hamad Medical Corporation Centre Laboratory, in Doha. Body composition was determined by Bioimpedance analysis (Tanita) and full body dual energy X-ray absorptiometry (iDXA) scan (GE healthcare, Illinois, USA). Body mass index (BMI) was calculated as weight in kilograms divided by height in meters squared (kg/m^2^). Obesity was defined as BMI ≥ 30 kg/m^2^. Fat mass index (FMI) was calculated by dividing fat mass in kilograms by height in meters squared (kg/m^2^). Body adiposity index (BAI) was calculated using the formula: BAI = [((hip circumference (cm)/ (height (m)^1.5^))] − 18^47^.

### Quantification of the salivary α-amylase activity in plasma

The quantification of the plasma sAA (psAA) was estimated by an enzymatic colorimetric assay with an autoanalyzer (ARCHITECT c4000; kits # 6K22-30 and #7D58-21; ABBOTT laboratories, Bluff, Illinoi, USA). More details about the assay can be found in supplementary material.

### Estimation of AMY1 CNVs

The AMY1 gene CN was estimated from whole-genome sequencing data and CNVnator version 0.4, which uses read-depth (RD) analysis of genome sequencing for CNV discovery and genotyping^48^. Briefly, Chr1-aligned reads were extracted from the WGS bam files and indexed using Bamtools^49^. Read mappings were then extracted and the RDs calculated for regions of 1 kb using CNVnator. The reads were partitioned into non-overlapping 100 base regions, which were then normalized. CNV counts were then estimated from the normalized read counts and filtered for regions of interest. Sample CNV counts were then estimated by summing the counts for AMY1A specific regions of interest. The copy number of AMY1 was rounded to the nearest integer.

### Statistical analysis

Statistical analyses were carried out with Stata 15.1/IC software (https://www.stata.com). Descriptive statistics were used to present the data mean and standard deviation. Comparison of baseline characteristics between groups was performed with independent samples t-test. For categorical variables and proportions Chi^2^ test was applied. Variables with outliers were winsorized using winsor2 command in Stata. Correlations were tested with Pearson coefficient. Linear regression was used to investigate associations in more detail and β-coefficient was used to quantify the association. Adjusted logistic regression was used to assess the association of psAAa or AMY1 gene CN with rate of obesity. A *p* value < 0.05 was considered statistically significant. None of the variables had more than 7% missing values, which were imputed using multiple imputations by chained equations in Stata.

## Supplementary information

Supplementary Information.

## Data Availability

Clinical, anthropometric, demographic and genetic data can be obtained from the Qatar biobank according to the applied rules.

## Abbreviations

BAI: Body adiposity index
BMI: Body mass index
BW: Body weight
FMI: Fat mass index psAAa
MetS: Metabolic syndrom
psAAa: Plasma salivary alpha-amylase activity
sAA: Salivary alpha-amylase
T2D: Type 2 diabetes
VAT: Visceral adipose tissue
WHR: Wait-to-hip ratio
