# Supplementary material for: High plasma salivary α-amylase, but not high AMY1 copy number, associated with low obesity rate in Qatari adults: cross-sectional study
Source: Sci Rep. 2020 Oct 21;10:17918. doi: 10.1038/s41598-020-74864-6 (PMC7578794; doi:10.1038/s41598-020-74864-6)
Supplement: Supplementary file 1 — Supplementary Information. [file 41598_2020_74864_MOESM1_ESM.docx]

**Supplementary material**

**High plasma Salivary α-amylase, but not high AMY1 copy number, associated with low obesity rate in Qatari adults: cross-sectional study**

Nayla S. Al-Akl^1^, Richard I. Thompson^1^, Abdelilah Arredouani^1*^

^1^ Qatar Biomedical Research Institute/ Hamad Ben Khalifa University, Education City, Doha, Qatar

**Methods**

**Quantification of plasma sAA activity**

The quantification of the plasma sAA activity (psAAa) was estimated by an enzymatic colorimetric assay with an autoanalyzer (ARCHITECT c4000; kits # 6K22-30 and #7D58-21; ABBOTT laboratories, Bluff, Illinoi, USA). The procedure consists of two reactions to measure the enzymatic activities of the total α-amylase (tAA) and the pancreatic α-amylase (pAA). The activity of the sAA is obtained by subtracting the activity of the pAA from that of the tAA. To obtain the pAA activity the assay is performed in two successive steps (kit # 6K22-30). In the first step, the activity of the sAA is inhibited using two different monoclonal antibodies with no effect on the pAA. In the second reaction step, the pAA catalyzes the hydrolysis of the EPS substrate (Ethydien Protected Substrate) p*-*Nitrophenyl*-*maltoheptaoside 4,6-ethylidene-blocked (ethylidene-G7PNP) forming 2 ethylidene-G5 + 2 G2PNP + 2 ethylidene-G4 + 2 G3PNP + ethylidene-G3 + G4PNP. The α-glycosidase hydrolyzes all the fragments of the G2PNP, G3PNP, and G4PNP into phenol (PNP) and glucose (G). The increase of absorbance due to PNP formation is proportional to the activity of pAA in the examined sample. For the tAA activity, α-amylase hydrolyzes the 2-chloro-4-nitrophenyl-α-D-maltotrioside (CNPG3) substrate to release 2-chloro-4-nitrophenol (CPNP) and form 2-chloro-4-nitrophenyl-α-D-maltoside (CNPG2), maltotriose, and glucose. The rate of formation of the 2-chloro-4-nitrophenol can be detected spectrophotometrically at 404 nm to give a direct measurement of the total α-amylase activity in the sample. Plasma sAA enzymatic activity was calculated by subtracting the activity of the pAA from the activity of tAA (psAA = tAA-pAA).

**Results**

| **Table S1** Correlations between psAAa or AMY1 CN and adiposity markers by gender  r: Pearson coefficient. Significant p values at 5% level are in bold | | | | | | | | |
| --- | --- | --- | --- | --- | --- | --- | --- | --- |
| Variables | **Plasma sAA activity** | | | | **AMY1 gene CN** | | | |
|  | **Men (n=431)** | | **Women (n=498)** | | **Men (n=431)** | | **Women (n=498)** | |
|  | r | *p value* | r | *p value* | r | *p value* | r | *p value* |
| **BMI (Kg/m^2^)** | -0.24 | **<0.001** | -0.12 | **0.01** | -0.11 | **0.02** | -0.01 | 0.78 |
| **Waist (cm)** | -0.18 | **<0.001** | -0.09 | **0.03** | -0.08 | 0.08 | 0.01 | 0.75 |
| **Hip (cm)** | -0.19 | **<0.001** | -0.13 | **0.003** | -0.08 | 0.09 | -0.02 | 0.73 |
| **WHR** | -0.09 | 0.07 | -0.02 | 0.64 | -0.05 | 0.27 | 0.04 | 0.40 |
| **Fat Mass (kg)** | -0.18 | **<0.001** | -0.15 | **<0.001** | -0.12 | **0.01** | -0.03 | 0.52 |
| **FMI (Kg/m^2^)** | -0.21 | **<0.001** | -0.14 | **0.002** | -0.15 | **<0.001** | -0.03 | 0.53 |
| **BF (%)** | -0.18 | **<0.001** | -0.16 | **<0.001** | -0.13 | **0.01** | -0.05 | 0.25 |
| **BAI** | -0.17 | **<0.001** | -0.11 | **0.02** | -0.11 | **0.02** | -0.01 | 0.74 |
| **VAT (Kg)** | -0.14 | **<0.001** | -0.13 | **0.003** | -0.08 | 0.09 | -0.01 | 0.84 |
| **BW (Kg)** | -0.22 | **<0.001** | -0.12 | **0.005** | -0.08 | 0.11 | -0.01 | 0.86 |

| **Table S2** Association between psAAa or AMY1 gene CN and the rate of overweight/obesity (OW/OB) in men and women. Data are mean (+/- SD) or mean. The table includes the logistic regression analysis adjusted for age. Cases are the OW/OB individuals while the controls are the normal-weight individuals | | | | | |
| --- | --- | --- | --- | --- | --- |
| **Predictor variables** | **Men (n=431)** | | | | |
|  | Cases (n=328) | Controls (n=103) | OR | 95 % CI | *p* |
| **psAAa (U/L)** | 32.3 (12.89) | 36.1 (12.37) | 0.976 | 0.95-0.99 | **0.008** |
| **AMY1 CN** | 7 | 7 | 0.973 | 0.90- 1.05 | 0.496 |
|  | **Women (n=498)** | | | | |
|  | Cases (n=385) | Controls (n=113) | OR | 95 % CI | *p* |
| **psAAa (U/L)** | 29.3(11.78) | 32.47 (14.32) | 0.982 | 0.96-1.00 | 0.067 |
| **AMY1 CN** | 7 | 7 | 0.945 | 0.87- 1.02 | 0.162 |
